# Use of prothrombin complex concentrate in warfarin anticoagulation reversal in the emergency department: a quality improvement study of administration delays

**DOI:** 10.1186/s12913-015-0775-6

**Published:** 2015-03-15

**Authors:** Simon Bordeleau, Julien Poitras, Danièle Marceau, Carolle Breton, Pierre Beaupré, Patrick M Archambault

**Affiliations:** Emergency Medicine Training Program, Département de médecine familiale et médecine d’urgence, Université Laval, Québec, QC Canada; Centre de santé et de services sociaux Alphonse-Desjardins (Centre hospitalier affilié universitaire de Lévis), Lévis, QC Canada; Département de médecine familiale et médecine d’urgence, Université Laval, Québec, QC Canada; Blood bank director, Quebec, Province of Quebec, Région 12 Canada; Transfusion Safety Officer, Quebec, Province of Quebec, Région 12 Canada; Division de soins intensifs, Département d’anesthésiologie, Université Laval, Québec, QC Canada

**Keywords:** Quality improvement program, OMRU, Audit and feedback, Prothrombin complex concentrate, Administration delay, Warfarin, Anticoagulation haemorrhage reversal, Emergency department

## Abstract

**Background:**

Quick reversal of warfarin anticoagulation is important in life threatening bleeding. The aim of this study is to improve the administration delay when using Prothrombin Complex Concentrate (PCC) for the emergent reversal of warfarin anticoagulation in the emergency department.

**Methods:**

An audit and feedback quality improvement project was conducted in three phases: a retrospective audit phase, an analysis and feedback phase and prospective evaluation phase. The charts of all eligible patients in a single Emergency Department (ED) in Québec, Canada, who received PCC since the introduction of this product in 2009 until October 31, 2011, were retrospectively audited. The administration delay of PCC was calculated from the time of prescription to the time of administration. With the data, we determined where improvements could be attained, and jointly with all stakeholders in the ED and the blood bank, we created an action plan to ensure the timely administration of PCC. The action plan was then implemented and a six-month prospective evaluation study was conducted to determine any improvement.

**Results:**

Seventy-seven charts were reviewed in the retrospective chart audit. The mean administration delay was 73.6 minutes (STD [34.1]) with a median of 70.0 minutes (25–75% IQR [45.0–95.0]). We found that this delay was principally due to the following barriers: communication problems between the ED and the blood bank as well as delivery inefficiencies. An action plan that involved a flowchart to remind all clinicians how to order PCC and a new delivery method from the blood bank to the ED were developed. During the 6 months following the implementation of our action plan, 39 patients received PCC and the mean administration time decreased to 33.2 minutes (STD [14.2])(*p* < .0001) with a median of 30.0 minutes (25–75% IQR [24.3–38.8]).

**Conclusion:**

By implementing an action plan comprising of a flowchart and a new delivery process, this audit and feedback quality improvement project reduced the administration time of PCC by more than half. Future studies to measure the impact of a similar audit and feedback process involving an action plan in other centers should be conducted before this type of quality improvement process is implemented on wider scale.

## Background

Anticoagulation with oral warfarin is a common treatment for long-term prevention in cardiac, cerebral, pulmonary or lower limb thromboembolic events [1-3]. The use of this therapeutic modality is strongly recommended in the medical literature but its narrow therapeutic index and the bleeding risk associated with the therapy are often matters of concern [3]. In the event of major life-threatening bleeding, such as intracranial haemorrhage, delaying the reversal of anticoagulation increases mortality [4-6]. Thus, quick reversal of anticoagulation in these cases is mandatory.

Two blood products--fresh frozen plasma (FFP) and the new prothrombin complex concentrate (PCC)--are used in addition to vitamin K to reverse warfarin anticoagulation in emergent situations [3,7-14]. Several studies have recently shown the efficacy of PCC over FFP in the treatment of major bleeding caused by warfarin [8,9,14-18]. The delay between prescription of FFP and subsequent reversal of warfarin anticoagulation can be long and may take hours [19-22]. In contrast, PCC-mediated reversal of anticoagulation is achieved in only 10 to 15 minutes after administration [23,24]. In optimal setup, it can take around 20 minutes to prepare PCC and administer the product [25]. However, total administration times between 60 to 200 minutes are reported in the literature [7,20,26,27]. Shortening the time it takes to prepare and administer PCC is essential because controlling active bleeding sooner could lead to better clinical outcomes [4-6,28,29].

In clinical practice, strong quality improvement programs are important as new products are continuously implemented. Audit and feedback is one such quality improvement process that generally leads to small but potentially important improvements in professional practice [30]. Audit and feedback is widely used as a strategy to improve professional practice either on its own or as a component of multifaceted quality improvement interventions [30]. Professional practice and performance are audited and compared to standards published in clinical practice guidelines and other medical literatures. The results of the audit are then analyzed with the aim to give feedback to healthcare professionals so that they improve their performance. Providing feedback with the support of an action plan has been shown to improve its impact [30]. However, more evidence is needed about how to develop these action plans and to assess their impact on the quality of care [30]. Thus, we report our experience with the development of an action plan used to support an audit and feedback quality improvement project conducted to improve the administration of PCC for the reversal of warfarin in the context of life-threatening bleeding. The specific objectives of this quality improvement project were to analyse the use of PCC in our emergency department (ED); to develop an action plan to improve the administration delay of PCC in our center; and to prospectively evaluate the implementation of this action plan.

## Methods

### Study setting

This study was conducted at the CSSS Alphonse-Desjardins (CSSS AD) as a three-part study. The CSSS AD is a university-affiliated Level 2 trauma center that receives a volume of sixty-five thousand visits per year. Since PCC is a blood product, the blood bank stocks and manages its use. Of the two PCC products available in Canada (Octaplex®, Octapharma Canada [10] and Beriplex®, CSL Behring Canada [11]), Octaplex® was the one available at our center over the time of this study. PCC is provided in a powder format kept in vials at room temperature. When needed, PCC is reconstituted with sterile water in a 6-step procedure [25]. PCC administration requires no previous blood typing or cross-matching [25]. When PCC were first introduced at our institution in November 2009, a protocol for the prescription (indications, doses, contraindications) of PCC for emergency physicians was initially proposed by our blood bank haematologist and then approved at the transfusion medicine committee formed by an emergency physician, ward physicians, the blood bank safety officer and the blood bank haematologist [31]. Whenever the PCC protocol was ordered by an emergency physician, a written prescription was sent to the blood bank by an ED nurse. A blood bank technician then reconstituted the PCC after which an orderly was asked to deliver the product to the ED where a nurse then administered it. PCC is administered as an infusion at 1 cc per minute for 5 minutes then 2–3 cc per minute, with a general starting dose of 2 vials and a total infusion time of approximately 15 minutes for 2 vials (Octaplex®) [25]. For patients over 90 kg, a higher dose was indicated (maximum of 6 vials) [10,11,32].

### Conceptual framework

**Figure 1:**
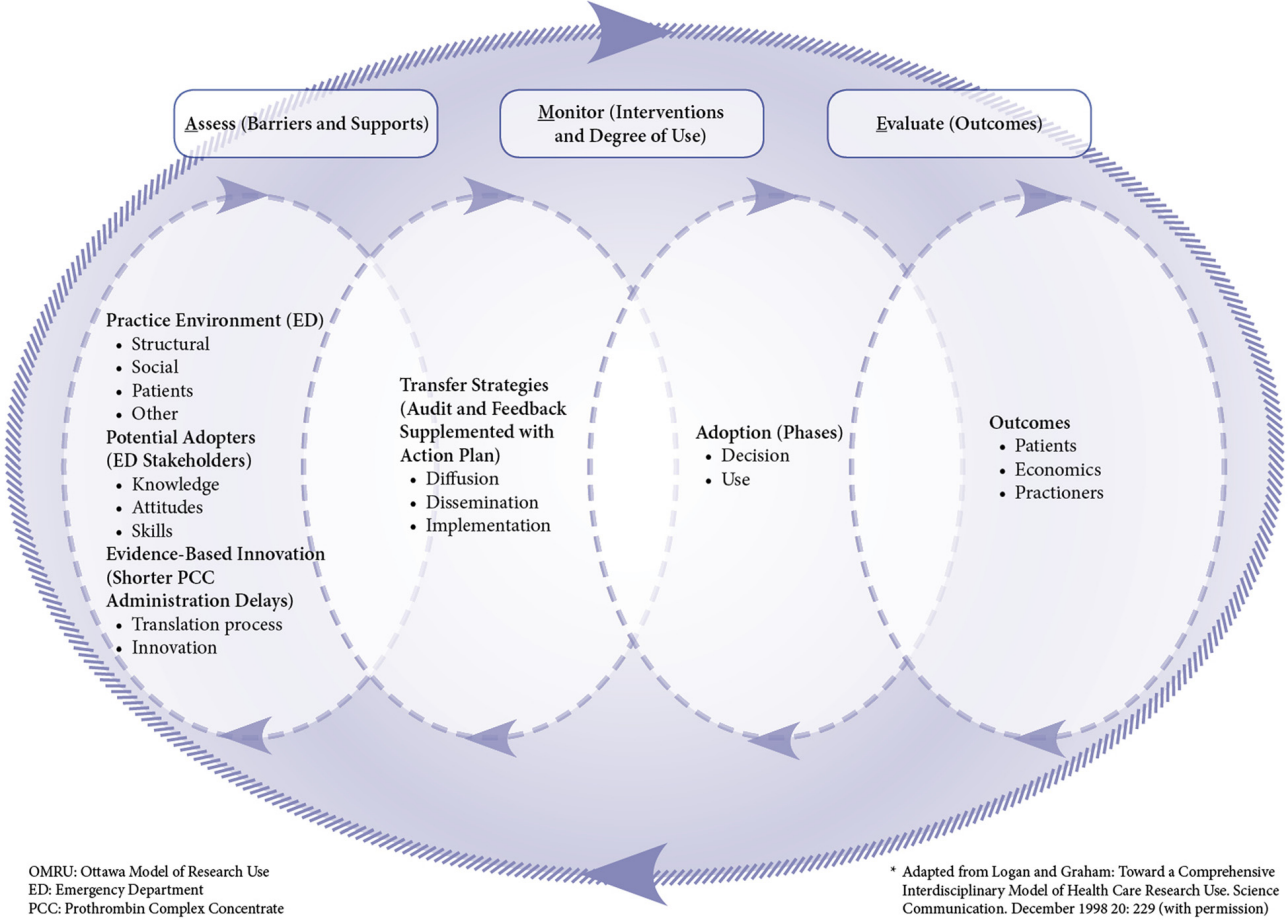
**OMRU applied to our quality improvement project.** OMRU: Ottawa Model of Research Use, ED: Emergency Department, PCC: Prothrombine Complex Concentrate. Adapted from Logan and Graham: Toward a Comprehensive Interdisciplinary Model of Health Care Research Use. Science Communication, December 1998 20:229 (with permission).

To help guide the development of our quality improvement project, we used the Ottawa Model of Research Use framework (OMRU) (Figure 1) [33,34].

This framework employs a 6-step method to guide the implementation of any evidence-based innovation. The first step involves setting the stage by identifying all the important stakeholders. Second, the innovation must then be specified with its implementation implications. Third, the OMRU calls for the conduct of an audit and feedback process to identify the current knowledge implementation gaps. Fourth, the OMRU proposes to select and implement knowledge translation strategies and then, as a fifth step, to monitor the adherence to these strategies. For the final step (sixth) in the OMRU framework, it is suggested to measure the clinical impact of implementing the knowledge innovation. In our project, the important stakeholders were the blood bank technologists, the blood bank haematologist and the emergency physicians, nurses and orderlies. Our innovation was the evidence about increased mortality associated with longer warfarin reversal and the need to decrease time delays.

### Phase 1: structured retrospective chart audit

The charts of all ED patients having received PCC between November 2009 and October 2011 were considered for inclusion. These charts were identified using a list of patients generated by the blood bank for all patients having received a prescription for PCC. Two reviewers assessed the inclusion of each chart and extracted the data using a structured extraction tool developed by the main author (Table 1).

**Table 1:** **Data collection tool**

**#**		
Age:	Gender:	Bleeding:
Date:	Time of prescription:	Administration time:
PCC:		
	Blood bank time:	
INR:		Time delay:

A chart was included only if the prescription of PCC was for the reversal of major bleeding in an ED patient anticoagulated with warfarin. Charts were excluded if the product was not administered in the ED, if no prescription time or end of infusion time were written in the chart or if the administration was cancelled. The following data was retrieved from the included charts: age, gender, time of prescription, start time of the reconstitution process, and end time of PCC infusion. We calculated the administration delay as the difference between the time the physician prescribed the blood product and the time at the end of the infusion, as noted by the nurse administering the PCC. A pre and post reconstitution time delay was calculated. The pre reconstitution time delay was the difference between the prescription time and the start of reconstitution of the product, as noted in the medical chart by the blood bank technician. The post reconstitution time delay was considered as the difference between the reconstitution starting time and the end of the infusion. We used simple descriptive statistics to present the demographic information (age, gender) of the included population and the means and medians for the different time delays measured.

This chart audit was approved by the Head of the ED who is responsible for our hospital’s quality improvement program managed by the Council of Physicians, Dentists & Pharmacists of the establishment (Québec, LSSSS, sec. 190). Under this jurisdiction, no approval from the institution’s ethics review committee is needed to perform such chart audits and after reviewing our full protocol, the ethics review committee (Comité d’éthique de la recherche du Centre de santé et services sociaux Alphonse-Desjardins (CER CSSS AD)) decided that our study was exempt from formal ethical approval.

### Phase 2: Identification of the barriers causing unwarranted time delay, development of an action plan to address these barriers and implementation of this action plan

After the completion of phase 1, the data was further analysed by the authors. The total administration time delay was broken down into its different components in order to identify the main sources of delay. Results of the chart audit were presented to all involved stakeholders. Through open discussion and group deliberation between authors and stakeholders, barriers to delivering PCC in a timely manner were identified. A comprehensive description of the current situation at our institution (i.e. results of the chart audit, our analysis of the causes for the different time delays, the barriers to timely PCC administration and the solutions proposed) was presented to the ED staff and to the transfusion committee for feedback. After this feedback was delivered, our research team and the stakeholders involved in this project jointly produced an action plan containing the strategies that were selected to improve PCC administration time delays and to offer solutions to the identified barriers.

Following the action plan development, the implementation was done in partnership with the blood bank, ED physicians and the nursing staff. A description of the important new steps in our developed action plan was also presented to all these stakeholders. This presentation included a 10 minutes in-service training session about the action plan. It was given to all concerned ED and blood bank personnel before its implementation. These training sessions were held by the transfusion safety officer and the emergency nurse educator. Moreover, these training sessions were financed by the institution. Finally, the action plan was also presented to all emergency physicians during a departmental meeting.

### Phase 3: Prospective evaluation of the developed action plan

Starting on June 1, 2012, all new cases of PCC prescription in the ED were subjected to the changes proposed by our action plan. Using the same data collection methods used in phase 1, data was collected for a six-month period between June 2012 and November 2012. Once the action plan was implemented, we prospectively collected data concerning administration delays in all cases of PCC administration in the emergency department. The transfusion safety officer collected the data immediately after each case. As in phase 1, patients were excluded if the product was not administered in the ED, if no time was written in the chart, or if the prescription was cancelled before administration. We collected data using the same structured data extraction tool employed in phase 1 (Table 1). However, we added ‘conformity with the new procedures’ proposed by our action plan as a new variable to extract. Non-conformity was considered if clinicians failed to perform any of the steps in our action plan. The reasons for non-conformity were noted, but patients were not excluded from the analysis. Similar to phase 1, we used simple descriptive statistics to present the demographic information of our sample (age, gender). We also calculated the mean and median administration delays. We used the same time delay definitions as phase 1. However, for this phase, we also conducted a comparative analysis between the retrospective cohort (before the action plan) and prospective cohort (after the implementation of the action plan) to assess the impact of our action plan.

### Statistical analyses

All the descriptive statistical analyses were performed with Excel 2007 (Microsoft Corporation, Redmond, WA) and the comparative statistical testing (Mann-Whitney *U* test) was conducted with a free online calculator (http://www.vassarstats.net/utest.html).

## Results

### Phase 1: structured retrospective chart audit

**Figure 2:**
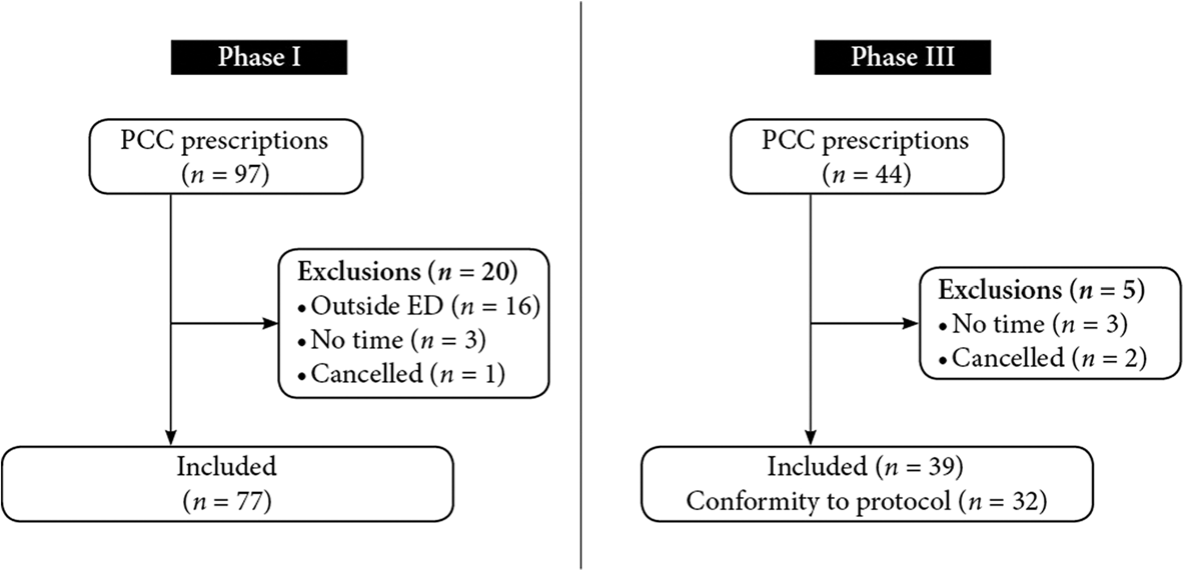
**Patients flowchart.**

In all, 97 adult patients were ordered to receive PCC between 2009 and 2011. However, the charts of twenty of these patients were excluded for the following reasons (Figure 2): 1) not an emergency room prescription (*n* = 16); 2) no administration time information charted (*n* = 3); and 3) cancelled prescription (*n* = 1).

Thus, our retrospective audit was conducted with 77 charts. Simple demographic information on included patients is reported in Table 2.

**Table 2:** **Demographic information**

**Phase 1**	
Number of patients	77
Age (Y)	80.5
Male sex	47%
**Phase 3**	
Number of patients	39
Age (Y)	75.8
Male sex	55%

The mean administration delay was 76.3 minutes (STD [34.1]) with a median of 70 minutes (25–75% IQR [55.0–95.0]) (Table 3).

**Table 3:** **Results**

**Time**	**Phase 1**	**Phase 3**	**P value**
Mean administration time (min) [STD]	73.6 [34.1]	33.2 [14.2]	<.0001
Median administration time (min) [25–75% IQR]	70.0 [55.0–95.0]	30.0 [24.3–38.8]	
Pre reconstitution median time (min) [25–75% IQR]	25.5 [12.8–34.5]	xxx	
Post reconstitution median time (min) [25–75% IQR]	43.5 [31.0–60.0]	xxx	
**Time**	**Phase 3 conform (82%)**	**Phase 3 Non-conform (18%)**	**P value**
Mean administration time (min) [STD]	28 [7.8]	56.9 [12.8]	0.0002
Median administration time (min) [25–75% IQR]	28.0 [24.0–33.5]	58.0 [56.5–65.0]	
Pre reconstitution median time (min) [25–75% IQR]	0 [0–0]	xxx	
Post reconstitution median time (min) [25–75% IQR]	28.0 [24.0–33.5]	xxx	

**Figure 3:**
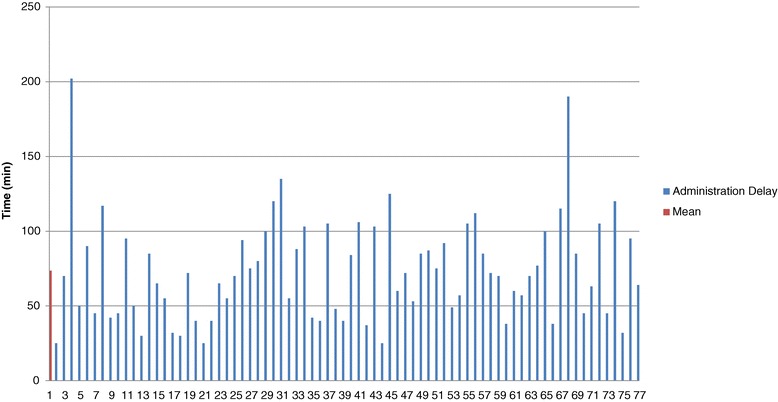
**Delay between prescription and administration of PCC – Phase 1.**

A large variation in delay measures was found between the cases (Figure 3). Only 36 charts had blood bank reconstitution starting times noted in the chart. With the available information in these 36 charts, the median pre reconstitution time was 25.5 minutes (25–75% IQR [12.8–34.5]). The median post reconstitution time was 43.5 minutes (25–75% IQR [31.0–60.0]).

### Phase 2: Identification of the barriers causing unwarranted time delays, development of an action plan to address these barriers and implementation of the action plan

**Figure 4:**
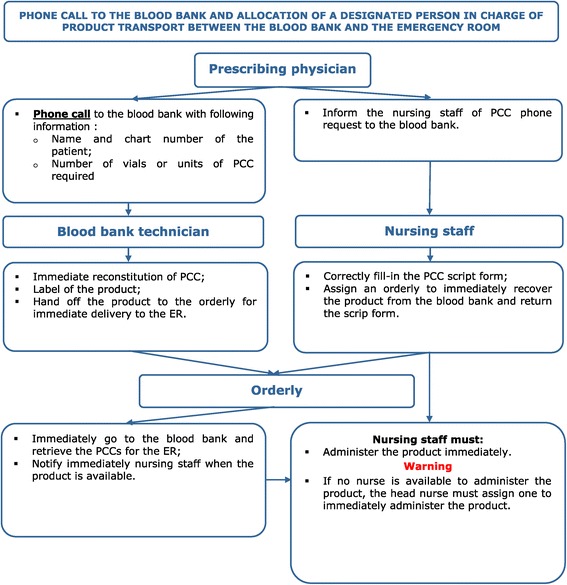
**Flowchart for the emergent administration of prothrombin complex concentrate (PCC) in the emergency department.** The goal of this chart is to reduce the delay to a minimum between the prescription and the administration of PCCs. We hope to achieve an optimal reversal of warfarin anticoagulation in patients with major bleeding.

The main barriers identified in phase 2 were the inefficient communication processes between the ED and the blood bank at the time of prescription, at the time of reconstitution and at the time of delivery of the reconstituted product from the blood bank to the ED. Several possible solutions to reduce the different time delays were debated (e.g. modifying the administration protocol and changing the site of reconstitution from the blood bank to the ED). A decision was then taken by the transfusion committee in partnership with the ED to review how PCC were prepared and delivered to the ED. This analysis of the delays and barriers resulted in the development of our action plan. The action plan proposed new procedures to reduce time delays caused by communication, reconstitution and delivery problems. During the development of our action plan, it was decided to maintain the responsibility of reconstituting PCC by blood bank technologists and not to transfer this task to ED nurses in order to maintain a central expertise for the whole hospital. In summary, our action plan involved these two following elements: a visual flowchart to remind all clinicians about the procedure to follow to order PCC from the blood bank (Figure 4) and a new delivery method to transport the ready-to-administer product from the blood bank to the ED.

The decision to ask ED physicians to call the blood bank directly rather than writing down an order and sending it to the blood bank was a major change. The visual flowchart was developed to inform and remind prescribing physicians of this change. It was also decided that ED physicians would be asked to advise the bedside nurse to assign an orderly to retrieve the prepared product immediately from the blood bank. Once the product was retrieved, a nurse had to administer it at once.

### Phase 3: Prospective evaluation of the newly developed action plan

**Figure 5:**
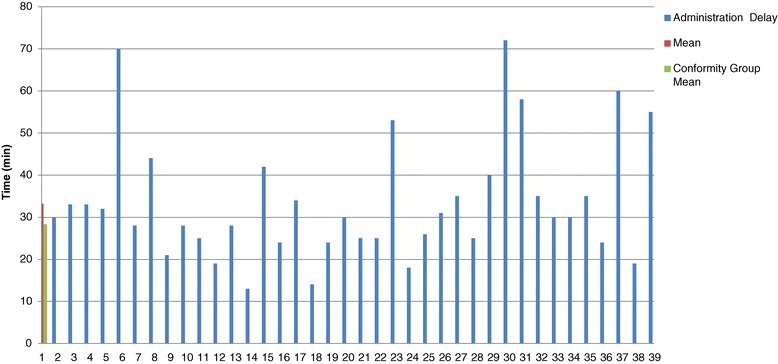
**Delay between prescription and administration of PCC - Phase 3.**

We prospectively collected information from 44 charts for six months. Five cases were finally excluded because no time information was recorded in the charts (*n* = 3), or PCC were never administered because the original order was cancelled (*n* = 2) (Figure 2). Thus, we analysed a total of 39 cases of PCC administration. Demographic and baseline information of these cases are found in Table 2. As in phase 1, all patients received Octaplex®. The mean administration delay was 33.2 minutes (STD [14.2]) and the median time was 30 minutes (25–75% IQR [24.25–38.75]). All of the new procedures proposed by the action plan (illustrated in the flowchart - Figure 4) were respected in 82% (*n* = 32) of cases analysed. The mean administration delay for these 32 cases was 28.0 minutes (STD [7.8]) (Table 3). Comparing our pre and post action plan time delays, the administration delay decreased from 73.6 minutes in phase 1 to 33.2 minutes in phase 3 (*p* < .0001). There was less variation in delay measurements in our prospective phase 3 cohort compared to our retrospective phase 1 cohort (Figures 3 and 5).

After reviewing the seven cases with the highest time delays in the prospective cohort, we noticed that all of them failed to conform to the procedures proposed in our action plan. Reasons for non-conformity included the physician not calling the blood bank (*n* = 2), nurses not sending an orderly to retrieve the product (*n* = 4) and nurses not administering the product immediately on reception (*n* = 1). The time delays for charts identified as not conform were longer than for the charts that were conform to the new procedure (mean administration time 56.9 vs. 28.0 minutes (p = .0002)) (Table 3). For the 32 cases that respected the newly implemented procedures, the median pre reconstitution time was 0 minute (25–75% IQR [0–0]) and the median post reconstitution time was 28.0 minutes (25–75% IQR [24.0–33.5]).

## Discussion

We performed an audit and feedback quality improvement project supplemented with an action plan to improve the administration delays of PCC in our center. Our chart audit found that our administration delays were high and could be improved. With the help of the important stakeholders, we developed our action plan to address the barriers we identified in administering PCC quickly and safely. These barriers concerned mostly communication breakdowns and difficult care processes regarding reconstitution and delivery of PCC from the blood bank. Our action plan involved providing understanding and training to all concerned personnel.

After implementing our action plan, we observed a significant decrease in the administration time delays. By comparing the pre and post reconstitution time delays for both our cohorts, we were able to confirm that our quality improvement project had a positive impact. One of the major issues we identified in phase 1 and 2 was the long pre reconstitution time delay that we believed was due to a communication breakdown between the ED and the blood bank. Part of our action plan was to ask ED physicians to call the blood bank directly to order PCCs instead of simply writing an order. After implementing this change, there was a reduction in this time delay.

As for the reconstitution of the product, it was decided to maintain the responsibility of preparing PCC by blood bank technologists as opposed to having ED nurses prepare the product in the ED, because PCC are blood products and reconstitution involves a few critical steps that benefit from having the expertise of a blood bank technician [25]. Moreover, concentrating this expertise in one place reduces the risk of errors and optimises reconstitution times for the whole hospital. We also believe that changing the way orderlies retrieved the prepared product improved the total administration delay. Our action plan involved immediately assigning an orderly to retrieve the product from the blood bank, even before it was ready, to minimise delivery time.

We also found that charts that were not conform to the action plan increased the administration delay. Without these events, we would have gained five more minutes in mean delay reduction (28.0 vs. 33.2 minutes). Since the goal of a quality improvement project is to reduce deviant practices, we believe that by providing regular in-house training, using a visual flowchart and other reminders could reduce the risk and the impact of these practices. A flowchart is a simple tool that communicates information visually and quickly. It requires little training to understand. Simple methods such as a flowchart can greatly improve administration, but adherence may be hindered by a variety of barriers [35]. In addition to the flowchart, all concerned personnel received a short in-service training. With this quality improvement project involving audit and feedback supplemented by an action plan, we improved our hospital’s performance with regards to PCC administration times in emergent situations. This reduction in PCC administration time accelerated the reversal of anticoagulation and helped clinicians stabilize their patients faster.

Even though our administration delays in phase 1 were comparable to those found in the literature before our feedback process [7,20,26,27], they were much higher than the optimal time [25]. However, after our audit and feedback process and the implementation of our action plan, our administration time decreased to 33 minutes which is close to the shortest administration delay possible.

This study has some limitations. We did not compare our intervention to another control intervention to serve as a point of reference. Thus, our positive findings could be influenced by a Hawthorne effect [36]. Moreover, we did not blind participants or outcome assessors. Thus, well-intentioned personnel seeking to improve their own performances could have charted better times of product prescription or end of administration times in phase 3. However, the fact that some of our measurements were charted by two different individuals (e.g. prescription times for PCC were charted both by ordering physicians and by the blood bank technologist) and that they did not differ makes this bias less likely. Also, our project did not verify if each prescription of PCC was written for a recognized indication or evaluate the appropriateness of PCC prescriptions. However, in order to minimise inappropriate usage and prior to phase 3, we updated our protocol (revised by our blood bank haematologist and then approved at the transfusion medicine committee) for PCC prescription to include a complete list of appropriate indications, the correct dosing regimen and the important contraindications [31]. We also reviewed this protocol with the emergency physicians during the presentation of our action plan.

Our research did not look at some of the delays that may occur before or after the prescription of PCC. Time delays caused by obtaining CT scans before PCC administration in case of intracranial haemorrhage can significantly slow down the process of reversing anticoagulation in this sub-group of patients [37] and reduce any gains due to our protocol. Time needed to obtain an INR may also delay the administration of PCC, given that the dose of PCC is dependent on the INR [38]. Rapid INR testing at the ED or PCC dosing independent from the INR may avoid this delay in emergent situations [31,38,39]. Lastly, administration time may vary between 15 to 30 minutes depending of the volume given, which depends on the weight, the INR, and the maximum infusion time specified by the manufacturer [25,32]. It has been shown in some studies that infusion time around 5 minutes are safe without increasing the thrombotic risk therefore leading to further reduction of the time needed to achieve anticoagulation reversal [38,40].

Notwithstanding these limitations, we believe that our quality improvement project describes how we developed a novel action plan to help implement the changes needed to improve PCC administration times. Audit and feedback are more effective when baseline performance is low, when the source is a supervisor or colleague, if it is provided more than once, if it is delivered in both verbal and written formats, and when it includes both explicit targets and an action plan [30]. Our study confirms that audit and feedback supplemented with a tailored action plan improved patient care by cutting the PCC administration delay by more than half in anticoagulated patients with warfarin presenting with major bleeding. Concerning our use of the OMRU, we also believe that it was one of the key elements to help us understand how our action plan helped us reduce the administration time of PCC in our institution. The OMRU helped us conceptualise how our action plan needed to be implemented. It helped us identify and involve the important stakeholders to develop an action plan that they would adopt. It also helped us identify the implementation barriers to our action plan and select the appropriate strategies adapted to our milieu to address these barriers. Finally, it helped us to monitor the adoption of our action plan and measure its impact.

Even though our quality improvement project was able to cut administration times, a local audit will be held each year at our institution to assess if our positive results are maintained. The impact of our quality improvement process performed in other centers and settings should also be studied to confirm our results. Although, it seems reasonable to believe that reducing PCC administration time could improve patient outcomes by controlling bleeding faster, future studies must also be conducted to measure the impact of this reduction on outcomes such as mortality and appropriate use of PCC.

## Conclusion

Warfarin anticoagulation reversal in life-threatening bleeding situations must be quick and performed without delay. New products are available but an efficient administration process is warranted in the emergency department. An audit and feedback process involving the development and use of an action plan summarized on a flowchart reduced the administration time of PCC by more than half. This process optimised the administration of PCC in emergent situations and reduced anticoagulation reversal delays in our ED. Although further improvements to reduce overall treatment time could make our action plan even more useful, an assessment of its impact on patient outcomes is warranted.

## Abbreviations

PCC: Prothrombin Complex Concentrate
ED: Emergency department
CSSS AD: CSSS Alphonse-Desjardins
OMRU: Ottawa Model of Research Use framework
